# Biological and Genomic Characterization of Two Astaxanthin-Producing *Paracoccus marcusii* Isolates as a Potential Source for Food Additives

**DOI:** 10.4014/jmb.2512.12023

**Published:** 2026-03-26

**Authors:** Keeman Lee, Eun Jeong Park, Jee Eun Han, Soojin Lim, Tae Seon Cha, Seunghui Lee, Seojeong Choi, Yejin Seo, Seon Young Park, Ji Hyung Kim

**Affiliations:** 1 Department of Food Science and Biotechnology, College of BioNano Technology, Gachon University, Seongnam 13120, Republic of Korea; 2 Institute for Veterinary Biomedical Science, Department of Veterinary Medicine, Kyungpook National University, Daegu 41566, Republic of Korea; 3 Laboratory of Aquatic Biomedicine, College of Veterinary Medicine and Research Institute for Veterinary Science, Seoul National University, Seoul 08826, Republic of Korea; 4 Veterinary Drugs and Biologics Division, Animal and Plant Quarantine Agency, Gimcheon 39660, Republic of Korea; 5 Department of Veterinary Medicine, Faculty of Veterinary Science, Chulalongkorn University, Bangkok 10330, Thailand

**Keywords:** Carotenoid biosynthesis, Antioxidant activity, Microbial pigment, *crtWZYIBE*

## Abstract

Astaxanthin (AST), a carotenoid pigment, has garnered significant interest due to its potent antioxidant, anti-inflammatory, and antibacterial properties, indicating that it is a valuable natural additive in the aquaculture, nutraceutical, and cosmetic industries. To date, *Paracoccus* spp., a known astaxanthin-producing bacteria, has emerged as a potential microbial source of substantial AST production yield and biosynthetic capabilities. This study reports the biochemical and genomic characterization of two *Paracoccus* isolates, GCUPA1 and GCUPA3, focusing on their potential as sources of natural carotenoids. Both strains were characterized by distinctive red-orange pigmentation and identified as *P. marcusii* based on 16S rRNA analysis. Spectroscopic and chromatographic analyses were performed to identify the predominant carotenoids, and the results established AST as the predominant carotenoid in both strains. The extracted pigments exhibited significant antioxidant activity in the 2,2′-azino-bis (3-ethylbenzothiazoline-6-sulfonic acid) (ABTS) assay, indicating their potential to reduce oxidative stress. Genome phylogeny revealed that both strains were closely related to the carotenoid-producing strain, *P. marcusii* CP157, confirming their taxonomic position within the species. Notably, the complete genome sequences revealed intact carotenoid biosynthetic gene clusters (BGCs) that encode all essential enzymes (*crtWZYIBE*) required for astaxanthin synthesis from isoprenoid precursors, with high nucleotide identity between strains. These findings establish *P. marcusii* GCUPA1 and GCUPA3 as a potential cell factory for sustainable astaxanthin production and suggest significant advantages in terms of processing efficiency and production economics compared to existing microbial systems.

## Introduction

Astaxanthin (3,3′-dihydroxy-β, β-carotene-4,4′-dione, AST) is a red-orange ketocarotenoid with exceptionally high antioxidant activity, which is approximately 10-fold higher than that of other carotenoids and 100-fold greater than that of α-tocopherol [1]. AST has traditionally been obtained from marine organisms such as shrimp and krill, the microalgae *Haematococcus* (*H.*) *pluvialis*, and the yeast *Xanthophyllomyces dendrorhous* [2-4]. *H. pluvialis* currently accounts for most commercial production, accumulating up to 4% of its dry cell weight [5, 6]. However, this microalgal system has significant limitations, including prolonged cultivation periods exceeding 30 days, challenging extraction procedures due to its robust cell walls in the encysted phase, and reduced bioavailability of encapsulated pigment [7, 8], which have necessitated investigation into alternative biological production systems with enhanced efficiency and reduced processing complexity.

The mechanistic basis of the biological activity of AST involves multiple cellular pathways. AST inhibits the NF-κB, ERK1/2, and p38/MAPK signaling cascades while promoting activation of the PI3K/Akt and Nrf2 pathways, modulating the inflammatory response and oxidative stress resistance [9], and providing photoprotection against UV-induced damage by reducing matrix metalloproteinase expression and suppressing the expression of inflammatory mediators [10, 11]. Compared with microalgae, notable advantages are associated with the bacterial synthesis of carotenoids, such as rapid growth kinetics, straightforward cultivation simplicity, and improved extraction efficiency [12]. The genus *Paracoccus* (*P*.) (*Alphaproteobacteria*, *Rhodobacteraceae*) has emerged as a promising candidate for industrial AST production due to its metabolic versatility and carotenoid biosynthetic capabilities [13]. Several *Paracoccus* species, such as *P. haeundaensis*, *P. marcusii*, and *P. carotinifaciens*, synthesize various carotenoids via the action of biosynthetic gene clusters (BGCs), which encode the enzymes required for AST production from isoprenoid precursors [14]. Recent genomic analyses have revealed that these species harbor the complete BGCs required for carotenoid production, including the *crtW* and *crtZ* genes, which are essential for AST biosynthesis [15]. Additionally, these bacteria possess thinner peptidoglycan cell walls than microalgal systems, facilitating pigment extraction while maintaining production yields [16]. *Paracoccus* species demonstrate remarkable physiological plasticity and thrive across diverse environmental parameters, including temperatures ranging from 4–37°C, pH values of 4–10, and varying nutrient concentrations, eliminating the need for specialized growth media characteristics in microalgal cultivation [17, 18].

Despite the recognized potential of the *Paracoccus* species for biotechnological applications, characterization of their AST production capabilities remains limited. Previous studies have demonstrated carotenoid production in *P. haeundaensis* and *P. carotinifaciens* under specific cultivation conditions [14, 19]. However, the cultivation parameters, extraction methodologies, and biological activities of the carotenoids from *P. marcusii* strains have not been systematically evaluated. Additionally, biosafety considerations remain paramount because *P. yeei* has been identified as an opportunistic pathogen in immunocompromised individuals [20]. Therefore, we isolated and characterized two AST-producing *P. marcusii* strains, GCUPA1 and GCUPA3, from freshwater environments with the aims of: i) optimizing the cultivation conditions and effective extraction methodologies for carotenoids, ii) characterizing the BGCs responsible for AST production, iii) evaluating the antioxidant properties of the extracted pigments, and iv) assessing the safety of both strains for biotechnological applications.

## Materials and Methods

### Bacterial Isolation and Identification

Pigment-producing bacteria were isolated from freshwater samples from the Geumgang River. Single-pigmented colonies were selected and streaked onto fresh tryptic soy agar plates (TSA; Difco, USA) for single isolates. Isolates were then subcultured on TSA plates or in tryptic soy broth (TSB; Difco, USA) at 25°C and subjected to morphological characterization, as previously described [19, 21]. The biochemical properties of the isolates were analyzed using an API 20E kit (BioMérieux Inc., France) following standard protocols [22, 23]. Hemolytic activity was evaluated by culturing the isolates on TSA supplemented with 5% sheep blood [21, 24], and the isolated bacterial strains were identified using 16S rRNA sequencing. Bacterial genomic DNA was extracted using a DNeasy Blood & Tissue Kit (Qiagen, Germany), according to the manufacturer’s instructions. The 16S rRNA gene was amplified using universal primers 27F and 1492R, and the resulting amplicons were sequenced at Macrogen Inc. (Republic of Korea) using the universal primers 785F and 907R. The obtained bacterial 16S rRNA sequences were then compared with the GenBank database using BLAST and further phylogenetically analyzed using MEGA 11 software [25] to identify the taxonomic position of the isolates.

### Optimization of Temperature and Carbon Sources for Bacterial Growth

To determine the optimal growth conditions for the isolates, growth kinetic analyses were performed at various temperatures and with different carbon sources. The effect of temperature on growth was evaluated by inoculating each isolate in TSB with an initial inoculum of 1% (v/v) as a starter culture at four different temperatures (4, 16, 25, and 37°C). The optical density was then measured at 600 nm (OD_600_) every 12 h for a total of 48 h using an OPTIZEN POP UV/Vis spectrometer (KLAB, Republic of Korea). To evaluate the effects of different carbon sources on bacterial growth, Luria–Bertani (LB) broth (Difco) (10 g/l tryptone, 5 g/l yeast extract, and 10 g/l NaCl) was supplemented with glucose, fructose, galactose, sucrose, xylose, sorbitol, rhamnose, arabinose, and starch. The growth of the strains was evaluated using OD_600_ values.

### Antibiotic Susceptibility Assay

The sensitivity of the isolates to various antibiotics was evaluated using a method previously described for *P. yeei* [24] with minor modifications. Bacterial suspensions were prepared from fresh overnight cultures grown on TSA at 25°C, as described above (approximately 10^8^ colony-forming units (CFU/ml)). The minimum inhibitory concentrations (MICs) were determined using MIC test strips on Muller-Hinton agar (MHA; Difco) plates with the following antibiotics: amikacin (0.015–256 μg/ml), gentamycin (0.06–1,024 μg/ml), tobramycin (0.064–1,024 μg/ml), ceftazidime (0.016–256 μg/ml), imipenem (0.002–32 μg/ml), piperacillin/tazobactam (0.016/4–256/4 μg/ml), and ciprofloxacin (0.002–32 μg/ml). The plates were then incubated at 25°C for 48 h, and the presence of inhibition zones around the MIC strips was observed. Since the species-specific interpretive criteria for *Paracoccus* spp. are not currently available, sensitivity or resistance was determined using the clinical breakpoints for *Pseudomonas* spp. and *Acinetobacter* spp., which are recommended by the European Committee on Antimicrobial Susceptibility Testing (EUCAST) for non-fermentative Gram-negative bacteria lacking species-specific criteria [24]. For the remaining antibiotics, the non-species-related pharmacokinetic/pharmacodynamic (PK-PD) breakpoints provided by EUCAST were applied.

### Antibacterial Activity Test

The antibacterial activities of the isolates were evaluated using a previously described method [26], with minor modifications. Antibacterial activity was tested against eight reference bacterial strains: *Enterobacter cloacae* ATCC 13047^T^, *Escherichia* (*E.*) *coli* ATCC 43895, *E. fergusonii* ATCC 35469^T^, *Salmonella* Enteritidis KCCM 12021, *Staphylococcus* (*S.*) *ureilyticus* ATCC 49330^T^, *S. warneri* ATCC 27836^T^, *Vibrio* (*V.*) *alginolyticus* ATCC 17749^T^, and *V. haveyi* ATCC 14126^T^, which were cultured in TSB or marine broth (Difco) at 37°C and shaken to obtain 10^7^ CFU/ml. The bacterial isolates were cultured in TSB at 25°C until 10^7^ CFU/ml was obtained. The reference strains of *Enterobacter*, *Escherichia*, *Salmonella*, and *Staphylococcus* were inoculated at TSB and spread onto TSA plates, and those of *Vibrio* were inoculated at 2.5% NaCl-supplemented TSB and spread onto TSA or 2.5% NaCl-supplemented TSA plates. Sterile paper disks were then placed on the plates, and a 10 μL aliquot of the culture was spotted onto each disk. The plates were then incubated at 25°C for 24 h, and the antibacterial activity of the isolates was determined by measuring the clear zones indicating inhibition around the disks. Zones larger than 1 mm were considered to be indicative of antibacterial activity.

### Cell Cytotoxicity Assay

The cytotoxicity of the isolates was evaluated against the HT-1080 human fibroblastic epithelial cell line using a WST-8 cell viability assay kit (BIOMAX, Republic of Korea), as previously described [27] with minor modifications. The HT-1080 cell line was selected as it is a well-studied model widely used for *in vitro* cytotoxicity of microbial strains [28]. Briefly, HT-1080 cells were cultured in RPMI 1640 medium (Welgene, Republic of Korea) supplemented with 10% (v/v) heat-inactivated fetal bovine serum (MEDIAN Life Science, USA) and 1% (v/v) penicillin-streptomycin solution (PS; Welgene, Republic of Korea) and maintained at 37°C with 5% CO_2_. For the cytotoxicity assay, HT-1080 cells were seeded into 96-well plates (SPL, Republic of Korea) at a density of 5 × 10^4^ cells per well in 100 μl of fresh medium without PS solution and incubated for 24 h to allow cell attachment. The bacterial isolates were then cultured to a concentration of 10^7^ CFU/ml and serially diluted in phosphate-buffered saline (PBS, pH 7.4; Biosesang, Republic of Korea) to prepare suspensions of 10^4^, 10^5^, and 10^6^ CFU/ml. After cell attachment, 10 μl of each bacterial suspensions were added to each well of the 96-well plates containing the HT-1080 cells, resulting in final bacterial concentrations of approximately 10^3^ to 10^6^ CFU/ml per well. PBS was used as a vehicle control, and a non-inoculated medium was used as a negative control. To assess potential bacterial interference, the bacteria-only control was included as the background control. Cell viability was assessed at 0, 12, 24, 36, and 48 h post-inoculation. At each time point, 10 μl of WST-8 reagent was added to each well and incubated for 2 h at 37°C. The absorbance was measured at wavelengths of 450 nm and 600 nm using the SpectraMax ABS microplate reader (Molecular Devices, USA). The difference between the two absorbance values was used to determine the level of cellular respiration in each well, as an indicator of cell viability.

### Pigment Extraction and Total Carotenoid Content Determination

To determine the optimal culture conditions for AST production, the isolates were cultured in 100 ml TSB with 5% inoculum, and pigment concentration was monitored daily for 5 days (1, 2, 3, 4, and 5 days) by measuring absorbance at 480 nm. For pigment extraction and characterization, putative pigments of the isolates were extracted as previously described methods [29] with minor modifications. Briefly, the isolates were cultured in 100 ml TSB with 5% inoculum for 5 days, and bacterial cells were harvested by centrifugation at 10,000 rpm for 10 min. A higher inoculum volume (5%) was used for pigment extraction to maximize biomass accumulation and pigment yield, compared to the 1% inoculum used for growth characterization assays. The supernatant was then discarded, and the pellets were weighed. Crude pigments were extracted using three solvents: acetone (Duksan, Republic of Korea), methanol (Sigma, USA), and dimethyl sulfoxide (DMSO; Sigma), and the pellets were resuspended in four volumes (w/v) of the solvents [21, 30-32] and incubated at room temperature for 24 h to obtain a pure extract. The suspensions were then centrifuged at 12,000 rpm for 5 min, and the pigmented supernatant was filtered using a 0.22 μm PVDF syringe filter (Jet Biofil, China). Carotenoids follow the Beer-Lambert law, which states that the absorbance of a material is proportional to its concentration [33]. Thus, the amount of pigment can be estimated by measuring the absorbance of extracts at the maximum absorption wavelength (λ_max_). The λ_max_ of AST is 480 nm, and the carotenoid absorption coefficients used for quantification were 2,100 for acetone and methanol, and 2,220 for DMSO [29, 34, 35]. To calculate the total carotenoid content (TCC), the absorbance of the extracts was measured at 480 nm using a UV/Vis spectrophotometer (OPTIZEN POP, KLAB, Republic of Korea) using Eq. (1):

$$\text{TCC}\left(\text{μg/g}\right)\text{=}\frac{{\text{A×V×10}}^{\text{4}}}{{\text{A}}_{\text{1cm}}^{\text{1\%}}\text{×P}\left(\text{g}\right)}$$ (1)

where A=absorbance at 480 nm, V=total volume of extract (ml), ${\text{A}}_{\text{1cm}}^{\text{1\%}}$=carotenoid absorption coefficient in respective solvent, and P=mass of sample (g).

### Concentration and Spectroscopic Analysis of Crude Extracts

The crude pigment extracts obtained from the isolates were then concentrated to assess their composition and purity, and to identify the carotenoid compounds present. The crude extracts were then transferred to a 1 L flask using a rotary evaporator (EYELA, Japan) and evaporated at 40°C under decompression until all solvent was removed. The resulting dried pigment extracts were then resuspended in 100% acetone to obtain the concentrated pigment solutions, and the UV-visible light absorption spectra of the concentrated pigments and the standard AST solution (Sigma) were measured using a UV/Vis spectrometer (OPTIZEN POP, KLAB) in the wavelength range of 350–800 nm to determine the maximum adsorption wavelength of the pigments. The concentrated pigment extracts were then stored at 4°C until further analysis.

### High-Performance Liquid Chromatography (HPLC) Analysis of Crude Pigment Extracts

To analyze the composition of the obtained crude pigment extracts, chromatographic separation was performed using an Ultimate 3000 HPLC system (Thermo Fisher) equipped with a C18 reverse-phase column (ZORBAX Eclipse XDB-C18, 4.6 × 250 mm, 5 μm particle size; Agilent Technologies, USA) and a UV-vis detector. Crude pigment extracts were filtered through a 0.45 μm PTFE syringe filter (Jet Biofil) before injection, and 20 μl aliquots of each sample were injected into the HPLC system. The mobile phase consisted of a gradient of acetone and water (v/v), starting with 100% acetone and then gradually decreasing to 50% acetone. The flow rate was maintained at 1.0 ml/min, and the column temperature was set to 40°C. The UV-Vis detector (Thermo Fisher Scientific) was programmed to measure absorbance at 480 nm, corresponding to the maximum absorbance of carotenoids [36, 37]. Chromatographic separation was performed for 30 min, followed by a 20-min runtime to ensure column equilibration. The retention times of the main peaks in each sample were then compared to identify the carotenoid compounds in the crude extract. AST (Sigma) was used to generate reference chromatograms, and pigments were identified based on retention times. Data analysis was performed using Chromeleon chromatography data system software (Thermo Fisher Scientific), and the relative abundances of the carotenoid compounds in the crude extracts were determined by comparing the peaks in the obtained chromatograms.

### ABTS and DPPH Radical Scavenging Assays

The antioxidant activity of the crude pigment extracts was evaluated using ABTS (2,2′-azino-bis ((3-ethylbenzothiazoline-6-sulfonic acid)) and DPPH (2,2-Diphenyl-1-icrylhydrazyl) radical scavenging assays, as previously described [21, 38], with minor modifications. For the ABTS assay, a 7.4 mM ABTS solution (Sigma) was prepared in 2.6 mM potassium persulfate (Sigma) and allowed to stand at room temperature in the dark for 16 h to generate ABTS radicals (ABTS*). The ABTS* solution was then diluted with distilled water to obtain an absorbance of 0.70 ± 0.02 at 734 nm, and 980 μl aliquots of the diluted ABTS* solution mixed with 20 μl of the crude pigment extract samples at various concentrations (6.25–50 μg/ml). The mixtures were then incubated at room temperature in the dark for 10 min before the absorbance of each solution was measured at 734 nm using a UV/Vis spectrometer (OPTIZEN POP, KLAB, Republic of Korea). ABTS inhibition (%) was calculated using Eq. (2):

$$\text{ABTS and DPPH radical scavenging acitivty (\%) =}\left(\frac{A_{blank}-A_{sample}}{A_{blank}}\right)\times 100$$ (2)

Similarly, for the DPPH assay, a 0.1 mM DPPH solution was prepared in methanol, and 980 μl aliquots of the DPPH solution were mixed with 20 μl of crude pigment extract samples at different concentrations (6.25–50 μg/ml) before incubating at room temperature in the dark for 30 min. The absorbance of each solution was then measured at 517 nm using a UV/Vis spectrometer (OPTIZEN POP, KLAB), and the percentage inhibition (%) of the DPPH radicals was calculated using the same formula. Ascorbic acid was used as the positive control in both assays. The IC_50_ values, which represent the concentration of the crude pigment extract required to scavenge 50% of the ABTS* and DPPH radicals, were determined by plotting the inhibition (%) against the sample concentration and interpolating from the resulting linear regression curve. All assays were performed in triplicate.

### Genome Sequencing and Genomic Analysis

Genomic DNA was extracted from the isolates using the QIAamp DNA Mimi Kit (Qiagen), and the extracted DNA was sequenced using a hybrid approach that combined the PacBio Sequel System (Pacific Biosciences, USA) with a 20-kb SMRTbell template library and the Illumina HiSeq X-10 platform (Illumina, USA).The TruSeq Nano DNA Library Prep Kit (Illumina) was used for library preparation. The long-read sequences generated by the PacBio Sequel System were assembled using the Microbial Assembly Application software (Pacific Biosciences), and the resulting assembly was polished using Illumina paired-end reads to improve the accuracy of the genome sequence. Genome annotation was performed using the NCBI Prokaryotic Genome Annotation Pipeline [39]. To analyze the exact taxonomic position of the isolates within the genus *Paracoccus*, a genome-based phylogenetic tree was reconstructed using the genomes available for representative species in the genus from the GenBank database using the Type Strain Genome Server (TYGS) [40]. Orthologous Average Nucleotide Identity (OrthoANI) analysis was performed to compare the genomes of the isolates with those of closely related *Paracoccus* species, and OrthoANI values were calculated using OAT software (v. 0.93.1) [41]. Orthologous gene clusters of each isolate at the genome level were then obtained using OrthoVenn3 [42] with the closest strain based on the results of the OrthoANI analyses. The functional distribution of genes in the genomes of the isolates was categorized into Clusters of Orthologous Groups (COGs) using eggNOG-mapper v.2 [43]. The pathway distributions of the isolates were searched using BLASTKOALA [44] based on the Kyoto Encyclopedia of Genes and Genomes (KEGG) database and categorized by functional groups. The presence of BGCs in the genomes of the isolates was then identified using antiSMASH 5.2 [45] to detect potential secondary metabolite biosynthetic pathways, including those involved in pigment production. The detected pigment-related BGCs were compared with those of the most similar strains based on phylogenetic analysis [46] and visualized using Easyfig v.2.2.5. The pigment biosynthetic pathway was detected based on the KEGG database using the KEGG Mapper [15]. Potential antimicrobial resistance (AMR) genes were searched using the Resistance Gene Identifier (RGI) v.6.0.3 and the Comprehensive Antibiotic Resistance Database (CARD) v.3.3.0 [47]. Potential virulence factors of the isolates were detected using the Virulence Factor Database (VFDB), based on the genus *Vibrio*. The *Vibrio* VFDB dataset was selected as it includes virulence-associated genes commonly identified in aquatic bacteria, such as hemolysins, secretion systems, and metalloproteases. The presence of prophage regions within the genomes was identified using Phigaro v.2.3.0 [48] and the PHAge Search Tool with Enhanced Sequence Translation (PHASTEST) [49]. In PHASTEST analysis, prophage regions are classified based on a completeness score: intact (score > 90), questionable (score 70–90), or incomplete (score < 70), where the score reflects the presence of essential phage structural and functional modules such as integrase, capsid, head, tail, and lysis-related genes, rather than confirmed inducibility or lytic activity. Circular genome maps of the isolates were visualized using the online genome annotation tool in the Pathosystems Resource Integration Center (PATRIC) on the Bacterial and Viral Bioinformatics Resource Center platform [50].

### Culture Deposition and Nucleotide Accession Numbers

*P. marcusii* strains GCUPA1 and GCUPA3 were deposited in the Korea Collection for Type Cultures (KCTC) under accession numbers KCTC 16151BP and KCTC 16152BP, respectively. The complete genome sequences of strains GCUPA1 and GCUPA3 were deposited in the GenBank database under the accession numbers GCUPA1: CP101810 (chromosome) and CP101811–CP101818 (plasmids), GCUPA3: CP175545 (chromosome) and CP175546–CP175550 (plasmids).

### Statistical Analysis

All experiments were performed in triplicate, and the results are presented as the means ± standard deviation (SD). Statistical analyses were conducted using SPSS (version 28.0; IBM Corp., USA). One-way analysis of variance (ANOVA) was used to determine significant differences among the groups, followed by post-hoc multiple comparisons using Tukey’s honest significant difference (HSD) and Scheffe’s tests, with a *p*-value of less than 0.05 (*p* < 0.05) considered statistically significant in all tests. Graphical representations of the data were constructed using GraphPad Prism 8.0.1 (GraphPad Software, USA).

## Results

### Isolation and Identification of Two Pigment-Producing *P. marcusii* Strains

**Fig. 1:**
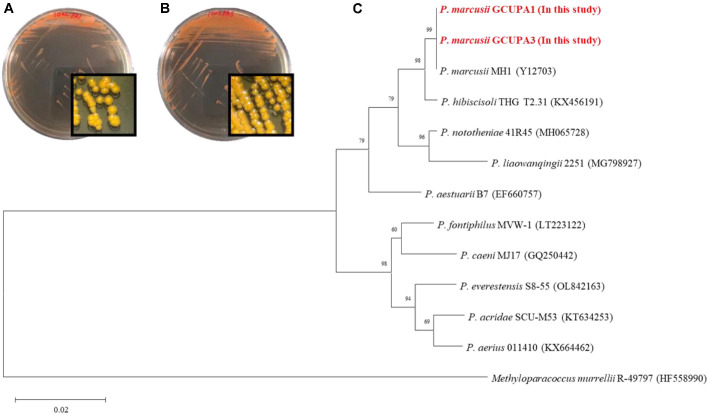
Morphological characteristics and 16S rRNA-based phylogeny of *P. marcusii* GCUPA1 and GCUPA3. (**A** and **B**) Colony morphology of strains GCUPA1 and GCUPA3 on TSA after 72 h incubation at 25°C, displaying the characteristic orange pigmentation. (**C**) Neighbor-joining phylogenetic tree based on 16s rRNA gene sequences showing the taxonomic position of strains GCUPA1 and GCUPA3 within the genus *Paracoccus*. Bootstrap values based on 1,000 replicates are shown at branch nodes.

Two bacterial strains, designated GCUPA1 and GCUPA3, were isolated from the freshwater samples and formed dense red-orange colonies on TSA plates after three days of incubation at 25°C (Fig. 1A and 1B). Both strains were non-hemolytic and positive for β-galactosidase activity and inositol production, but negative for other carbohydrate fermentation (Table S1), consistent with previously reported characteristics of *P. marcusii* [22]. Based on the 16S rRNA sequence analysis, the strains GCUPA1 and GCUPA3 showed > 99% sequence identity within themselves and were very similar (> 99.8%) to those of the *P. marcusii* strains available in the GenBank database. Additional phylogenetic analysis using the 16S rRNA gene against other representative species in the genus *Paracoccus* also revealed that GCUPA1 and GCUPA3 clustered well with the *P. marcusii* strain MH1^T^ (Y12703) (Fig. 1C). Based on these biological and genetic characteristics, the newly isolated strains, GCUPA1 and GCUPA3, were classified as *P. marcusii*. No hemolytic activity was detected in either strain (Data not shown).

### Optimal Growth Conditions for Two Pigment-Producing Strains, GCUPA1 and GCUPA3

**Fig. 2:**
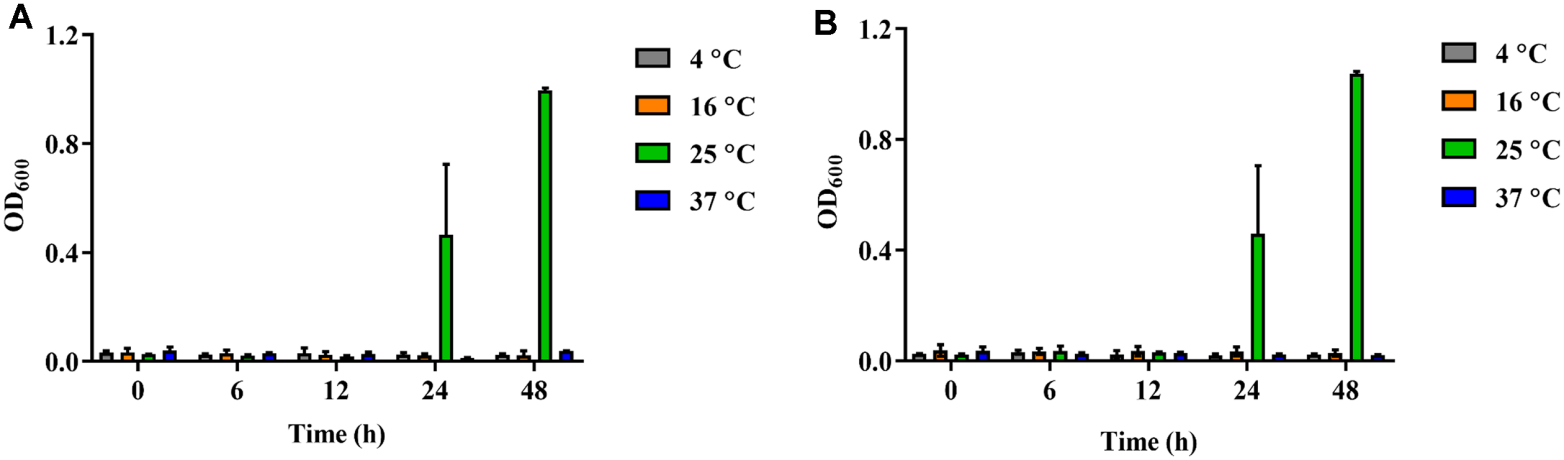
Temperature-dependent growth profiles of *P. marcusii* GCUPA1 (A) and GCUPA3 (B). Bacterial cultures were incubated at four different temperatures (4, 16, 25, and 37°C), and OD_600_ values were measured at 0, 6, 12, 24, and 48 h. All experiments were performed in triplicate, and error bars represent the standard deviation of the mean.

**Fig. 3:**
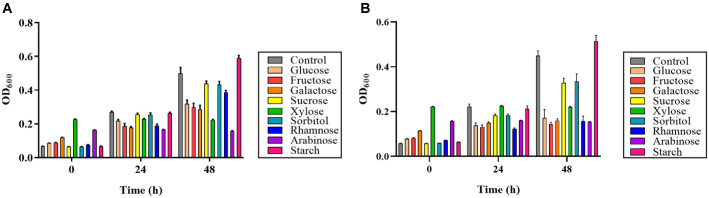
Effect of carbon source supplementation on growth of *P. marcusii* GCUPA1 (A) and GCUPA3 (B). Bacterial cultures were grown in modified LB broth supplemented with 1% (w/v) of different carbon sources: glucose, fructose, galactose, sucrose, xylose, sorbitol, rhamnose, arabinose, and starch. Growth was assessed by measuring OD_600_ after 24 h of incubation at 25°C. Control represents growth in unsupplemented LB medium. All experiments were performed in triplicate, and error bars represent the standard deviation of the mean.

The effects of temperature and carbon source supplementation on the *P. marcusii* GCUPA1 and GCUPA3 growth were evaluated over 48 h period, with measurements taken at 0, 6, 12, 24, and 48 h (Figs. 2 and 3). In terms of the temperature conditions, neither strain exhibited detectable growth at 4, 16, and 37°C after the 48-h observation period; however, both strains presented exponential growth at 25°C, with GCUPA1 reaching a maximum OD_600_ of 0.99 ± 0.01 and GCUPA3 achieving 1.04 ± 0.01 after 48 h of incubation, indicating that this temperature is optimal for the cultivation of these strains. Notably, despite the absence of fermentative activity for all the tested carbohydrates in the API 20E assay (Table S1), both strains exhibited differential growth conditions in response to the various carbon source supplements (Fig. 3). Strain GCUPA1 showed growth in the presence of glucose, fructose, galactose, sucrose, sorbitol, and rhamnose, with OD_600_ values decreasing to 0.29 ± 0.02 – 0.44 ± 0.01 as compared to the control (OD_600_ = 0.50 ± 0.03) after 48 h. In contrast, starch supplementation significantly enhanced GCUPA1 growth, with a 48 h-incubation OD_600_ of 0.59 ± 0.02, whereas xylose and arabinose had no significant effect on growth (OD_600_ = 0.22 ± 0.01 and 0.16 ± 0.01). Similarly, comparison of GCUPA3 with the control medium indicated growth inhibition for six of the nine tested carbon sources. In contrast, sucrose (OD_600_ = 0.32 ± 0.02), sorbitol (OD_600_ = 0.33 ± 0.03) showed reduced growth compared to the control (OD_600_ = 0.45 ± 0.02), while starch (OD_600_ = 0.52 ± 0.02) was the only supplement that significantly enhanced growth.

### Antibiotic Susceptibilities of Pigment-Producing Strains GCUPA1 and GCUPA3

The antibiotic susceptibility of *P. marcusii* GCUPA1 and GCUPA3 were determined using MIC tests on MHA plates, following the EUCAST guidelines (Table 1). Owing to the absence of species-specific breakpoints for *P. marcusii*, susceptibility was interpreted using previously established clinical breakpoints for *Pseudomonas* and *Acinetobacter* spp., along with non-species-related PK-PD breakpoints. Both strains demonstrated reactions to the majority of tested antibiotics, with MIC values within the sensitive ranges for aminoglycosides, β-lactams, and fluoroquinolones. However, both strains exhibited resistance to piperacillin/tazobactam, with MIC values exceeding 256/4 μg/ml. Strain GCUPA3 demonstrated higher sensitivities to amikacin (0.75 μg/ml), gentamicin (0.19 μg/ml), tobramycin (0.19 μg/ml), imipenem (0.094 μg/ml), and ciprofloxacin (0.016 μg/ml) as compared to GCUPA1. In comparison, GCUPA1 showed greater sensitivity to ceftazidime (1.5 μg/ml) and meropenem (0.023 μg/ml) as compared to GCUPA3.

### Antibacterial Activity of Strains GCUPA1 and GCUPA3

The antibacterial activity of *P. marcusii* GCUPA1 and GCUPA3 was assessed against eight potentially pathogenic bacteria using the disk diffusion method (Table 2). Neither of the strains exhibited inhibitory activity against seven of the tested pathogens: *E. cloacae* ATCC 13047^T^, *E. coli* ATCC 43895, *E. fergusonii* ATCC 35469^T^, *S. enteritidis* KCCM 12021, *S. ureilyticus* ATCC 49330^T^, *S. warneri* ATCC 27836^T^, and *V. harveyi* ATCC 14126^T^. However, *V. alginolyticus* ATCC 17749^T^ was susceptible to both GCUPA1 and GCUPA3, with clear inhibition zones (Table 2).

### Cytotoxicity Evaluation of Strains GCUPA1 and GCUPA3

**Fig. 4:**
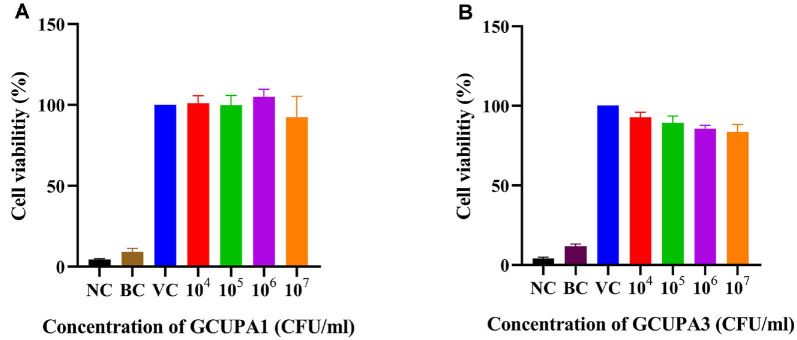
Cytotoxic effects of *P. marcusii* GCUPA1 (A) and GCUPA3 (B) on human fibroblast cells. HT-1080 cells (5 × 10^4^ cells/well) were exposed to different concentrations (10^4^ to 10^7^ CFU/mL) of the two strains for 24 h, and cell viability was determined using the WST-8 assay, with the results expressed as % relative to the untreated control (100%). All experiments were performed in triplicate, and error bars represent the standard deviation of the mean. Non-inoculated medium, bacteria only group, and PBS were used as the negative control (NC), background control (BC), and vehicle control (VC).

The potential cytotoxic effects of the *P. marcusii* GCUPA1 and GCUPA3 on human fibrosarcoma HT-1080 cells were also evaluated using the WST-8 cell viability assay (Fig. 4). To assess the cytotoxicity of the strains, both were tested at concentrations ranging from 10^4^ to 10^7^ CFU/ml in a dose-dependent manner. For strain GCUPA1, cells exposed to 10^6^ CFU/ml showed a slight increase in viability (105.6 ± 4.2%) as compared to untreated controls, whereas exposure to 10^7^ CFU/ml resulted in reduced cell viability (92.6 ± 11.6%). Similarly, GCUPA3 demonstrated cell viability values of 83.5 ± 1.8% at 10^6^ CFU/ml and 83.5 ± 4.1% at 10^7^ CFU/ml. The absence of significant cytotoxic effects at physiologically relevant bacterial concentrations indicates that both *P. marcusii* strains were non-pathogenic to human cells under the tested conditions.

### Pigment Extraction and Characterization

**Fig. 5:**
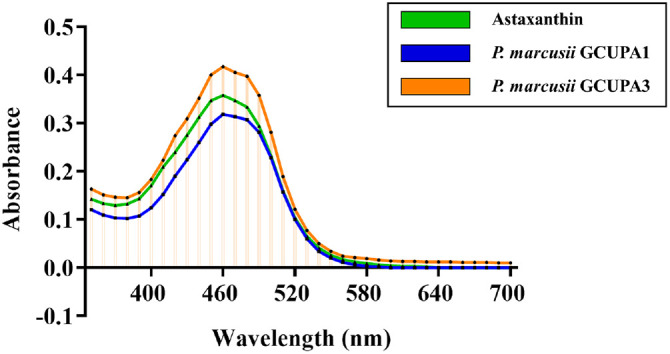
UV-visible absorption spectra of carotenoid pigments from *P. marcusii* GCUPA1 (blue line) and GCUPA3 (orange line). Spectrophotometric analysis was performed on concentrated acetone extracts from the two strains and compared with an authentic astaxanthin standard (green line). Absorption spectra were recorded from 300 to 700 nm using a UV-visible spectrophotometer. The astaxanthin standard exhibited a characteristic absorption maximum (λ_max_) at 460 nm. Both GCUPA1 and GCUPA3 extracts displayed similar spectral profiles, with λ_max_ at 465 nm, indicating carotenoid compounds with similar chromophore structures.

Pigments from *P. marcusii* GCUPA1 and GCUPA3 were extracted using three different solvents to optimize the extraction efficiency, with the acetone and methanol extracts yielding bright, orange-colored solutions, and the DMSO extract producing yellow-orange pigments. Notable differences in the cell pellet characteristics were observed post-extraction, with acetone- or methanol-treated pellets exhibiting dry, solid textures, indicating complete pigment removal, and DMSO-treated pellets retaining moisture and displaying an oily consistency. Quantitative analysis revealed that acetone provided the highest extraction efficiency for both strains (Table 3), with GCUPA1 yielding a total carotenoid content (TCC) of 64.1 ± 2.8 μg/g under acetone extraction, representing higher yields as compared to DMSO (33.5 ± 0.09 μg/g) and methanol (26.7 ± 1.7 μg/g) extraction. Similarly, strain GCUPA3 showed optimal extraction with acetone (45.2 ± 20.1 μg/g), followed by DMSO (22.6 ± 2.2 μg/g) and methanol (19.5 ± 5.7 μg/g). Acetone was selected for further characterization based on the extraction efficiency results. The concentrated acetone extracts from both strains showed a characteristic orange coloration with an oily consistency. UV-visible spectroscopic analysis revealed identical absorptions for GCUPA1 and GCUPA3, with a single maximum absorption peak (λ_max_) at 465 nm (Fig. 5). This absorption maximum is consistent with that of other carotenoid compounds, suggesting the presence of structurally similar carotenoids in both strains.

### HPLC Identification of Carotenoid Extracts

**Fig. 6:**
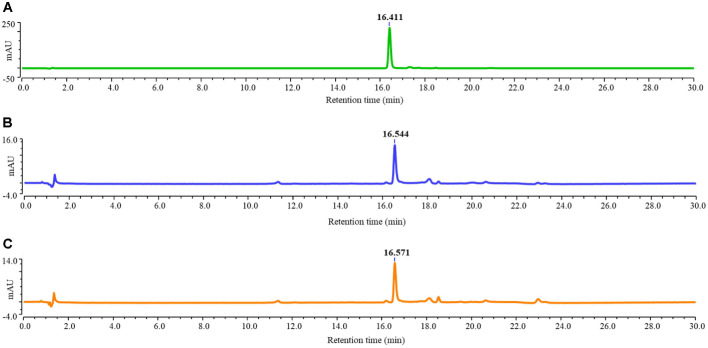
Reverse-phase HPLC chromatographic analysis of carotenoid extracts. Chromatographic separation was performed using a C18 column with an acetone:water gradient elution system at a 1 mL/min flow rate and detected at 480 nm. (**A**) Authentic astaxanthin standard showing a single peak at retention time (RT) 16.41 min. (**B**) Crude acetone extract from strain GCUPA1 exhibiting a predominant peak at RT 16.54 min. (**C**) Crude acetone extract from strain GCUPA3 displaying a major peak at RT 16.57 min. indicating similar to that of astaxanthin.

The carotenoid composition of the acetone extracts of *P. marcusii* GCUPA1 and GCUPA3 was determined by reverse-phase HPLC analysis and compared with the authentic AST standard. Chromatographic separation revealed distinct pigmentation features in both strains. The AST standard displayed a single major peak with a retention time (RT) of 16.41 min under the gradient conditions employed (Fig. 6A). Notably, the crude pigment extracts from the two strains demonstrated similar chromatographic features, with GCUPA1 exhibiting a predominant peak at an RT of 16.54 min (Fig. 6B) and GCUPA3 a major peak at an RT of 16.57 min (Fig. 6C) at 480 nm. The minimal deviation in the RT between the sample peaks and the AST standard (16.41 min) strongly suggests that the primary carotenoid AST or a structurally similar derivative was produced by both *P. marcusii* strains. The slight RT shift of 0.13–0.16 min may be attributed to a matrix effect resulting from co-extracted compounds in the crude extracts.

### Antioxidant Potential of the Carotenoid Extracts

To assess the antioxidant potential of *P. marcusii* GCUPA1 and GCUPA3, the radical-scavenging capacities of the pigment extracts from the two strains were evaluated using DPPH and ABTS assays. Both assays demonstrated concentration-dependent radical-scavenging activity, a previously reported characteristic that demonstrates the well-established antioxidant properties of carotenoid compounds [51, 52]. The results of the ABTS radical scavenging assay showed higher antioxidant activity for the GCUPA3 extract, with an IC_50_ value of 30.1 ± 1.4 μg/ml representing higher potency than the GCUPA1 extract, with an IC_50_ value of 53.1 ± 2.7 μg/ml. In contrast, the DPPH assay revealed better radical scavenging capacity for the GCUPA1 extract, with an IC_50_ value of 168.4 ± 22.4 μg/ml, as compared to the GCUPA3 extract with an IC_50_ value of 194.81 ± 11.95 μg/ml, indicating that both strains produce carotenoids with significant antioxidant properties.

### Genomic Characterization of Strains GCUPA1 and GCUPA3

**Fig. 7:**
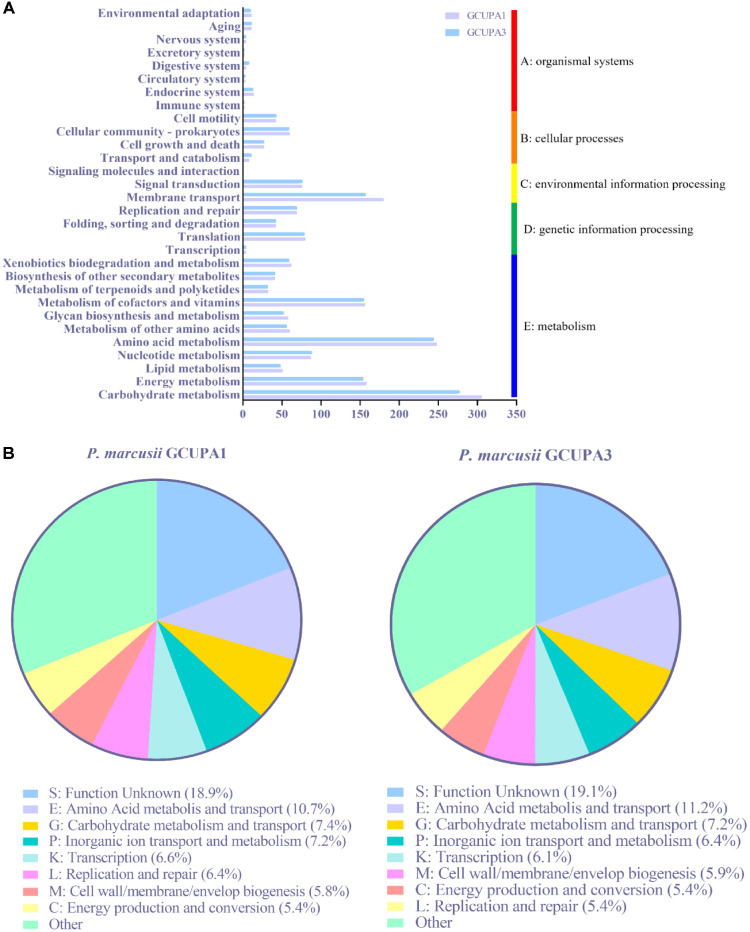
Functional genomic analysis of *P. marcusii* strains GCUPA1 and GCUPA3. (**A**) Distribution of protein-coding genes across Kyoto Encyclopedia of Genes and Genomes (KEGG) functional categories, determined using BlastKOALA with hidden Markov model searches against the KEGG GENES database. Functional categories are organized into five major groups: (a) Organismal systems, (b) Cellular processes, (c) Environmental information processing, (d) Genetic information processing, and (e) Metabolism. The number of genes assigned to each pathway is indicated. (**B**) Comparative analysis of Clusters of Orthologous Groups (COGs) distribution for strains GCUPA1 and GCUPA3 genomes using the egg Non-supervised Orthologous Groups (eggNOG)-mapper. Functional categories are represented as single-letter codes with relative abundance, shown as a percentage of the total annotated genes. Categories with < 5% representation were grouped as “Others.” Both strains exhibited similar functional features, with metabolism-related genes comprising the largest fraction of annotated sequences.

The sequenced GCUPA1 genome comprised 4,046,018 bp, with one circular chromosome (3,116,356 bp) and eight plasmids demonstrating an overall G+C content of 66.6% (Fig. S1). The genome encoded 3,922 protein-coding sequences (CDSs), 52 tRNAs, 9 rRNAs, 3 non-coding RNAs (ncRNAs), and 49 pseudogenes (Table S2). Similarly, the sequenced GCUPA3 genome comprised 3,446,361 bp, consisting of one circular chromosome and five plasmids with 67.3% G+C content (Fig. S2) that encoded 3,335 CDSs, 50 tRNAs, 9 rRNAs, 3 ncRNAs, and 16 pseudogenes (Table S2). The plasmid set differed notably between the strains, with GCUPA1 maintaining three additional plasmids (pGCUPA1_3, 255 genes; pGCUPA1_4, 198 genes; and pGCUPA1_5, 94 genes) encoding 547 CDSs involved in energy metabolism (pGCUPA1_3, 12 genes; pGCUPA1_4, 17genes; and pGCUPA1_5, 1 genes), transport (pGCUPA1_3, 11 genes; pGCUPA1_4, 20 genes; and pGCUPA1_5, 2 genes), transcriptional regulation (pGCUPA1_3, 16 genes; pGCUPA1_4, 11 genes; and pGCUPA1_5, 7 genes), and metal resistance (pGCUPA1_3, 6 genes; pGCUPA1_4, 3 genes; and pGCUPA1_5, 3 genes). Functional annotation via KEGG classification (Fig. 7A) revealed that metabolism was the predominant category in both genomes, comprising 32.1% (1,258/3,922) of the genes in GCUPA1 and 36.1% (1,206/3,339) in GCUPA3, followed by environmental information processing, which was observed in both strains, with 256 genes (13.5%) in GCUPA1 and 233 genes (12.8%) in GCUPA3. COG analysis with eggNOG5 demonstrated similar functional distributions between the strains (Fig. 7B), with enrichment observed for amino acid metabolism (E category: GCUPA1, 396 genes; GCUPA3, 361 genes), carbohydrate metabolism (G: GCUPA1, 277 genes; GCUPA3, 230 genes), and inorganic ion transport (P: GCUPA1, 270 genes; GCUPA3, 206 genes). This metabolic versatility was further evidenced by the presence of complete degradation pathways for aromatic compounds, including benzoate, catechol, protocatechuate, and gentisate, which is consistent with the environmental adaptability of *Paracoccus* species.

Both genomes harbored the complete carotenoid BGCs that are essential for AST production, with clusters encoding phytoene synthase (*crtB*), phytoene desaturase (*crtI*), lycopene cyclase (*crtY*), β-carotene ketolase (*crtW*), and β-carotene hydroxylase (*crtZ*), representing the complete enzymatic pathway for AST biosynthesis from isoprenoid precursors. Additionally, zeaxanthin glucosyltransferase genes suggest the capacity for glycosylated xanthophyll production. The carotenoid biosynthetic genes exhibited 100% nucleotide identity between GCUPA1 and GCUPA3, although different genomic arrangements were observed as compared to the reference strain CP157. Iron acquisition systems were well represented in both genomes, including complete TonB-ExbB/D complexes, multiple TonB-dependent receptors, and heme transport clusters (*ccmA*–*D* and *bhuA*), which facilitate growth under iron-limited conditions. Stress adaptation mechanisms included heavy metal tolerance proteins (*CzcO* and *ChrB*) and oxidative stress response systems, which is consistent with the ability of the strains to maintain carotenoid production under varying environmental conditions.

Secondary metabolite biosynthetic potential was demonstrated in multiple gene clusters. GCUPA1 contained a dimodular NRPS cluster and type I PKS-like modules on the plasmid pGCUPA1_4, whereas both strains encoded complete polyhydroxyalkanoate (PHA/PHB) operons, including the *phaCZPR* genes. Quorum-sensing capacity was confirmed by identifying the LuxI/LuxR homologs, suggesting the cell density-dependent regulation of metabolite production. Notably, both genomes contained complete pyrroloquinoline quinone biosynthesis (*pqqABCDE*) operons, which encode oxidative cofactors rarely reported in AST-producing bacteria. Safety assessments using CARD-RGI analysis revealed no clinically relevant antibiotic resistance genes in either genome. Virulence factor screening using the VFDB identified minimal pathogenicity determinants, limited to flagellar assembly proteins (*flhA* and *flmH*) and a type VI secretion system component (*clpV*), which are common in environmental bacteria (Table S3). Prophage analysis revealed four regions in GCUPA1 (two intact and two questionable) containing 77 phage-related genes, whereas GCUPA3 harbored three regions (two intact and one questionable) containing 55 phage-associated genes (Table S4). The absence of complete prophages and limited virulence factors supports the biosafety of both strains for biotechnological applications.

### Phylogenetic Analysis

**Fig. 8:**
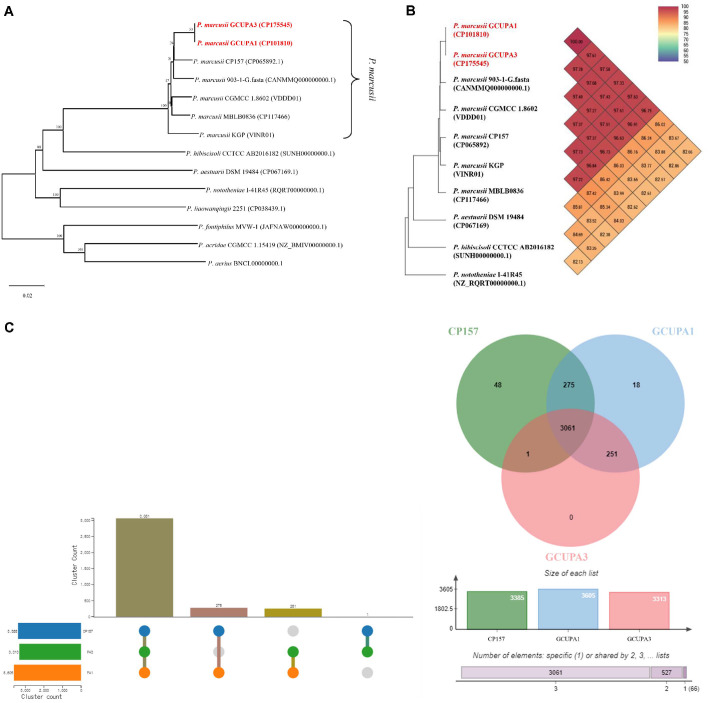
Phylogenetic and comparative genomic analyses of *P. marcusii* GCUPA1 and GCUPA3. (**A**) Genome-based phylogenetic construction of 16 *Paracoccus* species using the Type (Strain) Genome Server (TYGS) with Genome BLAST Distance Phylogeny (GBDP) analysis. (**B**) Heatmap showing the orthologous average nucleotide identity values calculated using OAT software. Strains GCUPA1 and GCUPA3 show > 97% ANI with representative *P. marcusii* strains, confirming species-level identity. (**C**) OrthoVenn diagram presenting the distribution of orthologous gene clusters among strains GCUPA1 (blue), GCUPA3 (red), and *P. marcusii* CP157 (green), generated using OrthoVenn3. The core genome comprises 3,061 gene clusters shared among all three strains, with strain-specific and pairwise-shared clusters indicated in respective regions.

GCUPA1 possesses 18 unique gene clusters and shares 275 clusters with CP157 and 251 with GCUPA3. Notably, GCUPA3 contained no strain-specific gene clusters and shared only a single unique cluster with CP157, indicating strain-level genomic diversity for the species (Fig. 8C). Genome-based phylogenetic analysis revealed that strains GCUPA1 and GCUPA3 clustered within a well-supported monophyletic clade that included six *P. marcusii* strains of the 14 representative *Paracoccus* species examined (Fig. 8A). Calculation of the orthologous average nucleotide identity (OrthoANI) values for the newly isolated strains and the closely related *Paracoccus* genomes (Fig. 8B) demonstrated the highest genomic similarity between GCUPA1 and GCUPA3 and *P. marcusii* 903-G-1.fasta (97.61% and 97.78%, respectively), followed by *P. marcusii* CGMCC 1.8602 (97.58% and 97.68%) and *P. marcusii* CP157 (97.33% and 97.43%). These ANI values exceeded the 95–96% threshold for species delineation, confirming their classification as *P. marcusii*. Pan-genome analysis of *P. marcusii* strains GCUPA1, GCUPA3, and the reference strain CP157 identified 3,061 core gene clusters conserved across all three genomes, representing approximately 78% of the total gene content (Fig. 8C). Strain-specific variations were evident in the genome, with GCUPA1 harboring 18 unique gene clusters that were absent in both GCUPA3 and CP157, whereas 275 gene clusters were shared exclusively with CP157 and 251 clusters with GCUPA3. Remarkably, GCUPA3 displayed minimal genomic novelty with no strain-specific gene clusters and shared a single unique cluster with CP157.

### Biosynthetic Gene Cluster Analysis

**Fig. 9:**
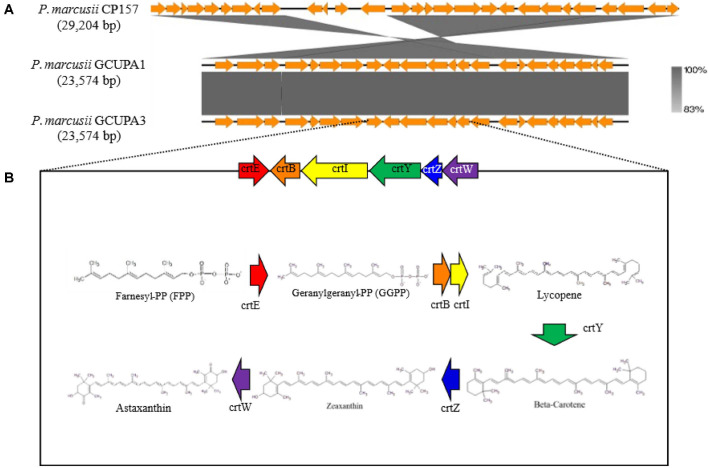
Organization and functional analysis of astaxanthin biosynthetic gene cluster in *P. marcusii* strains. (**A**) Comparative visualization of carotenoid biosynthesis (*crt*) gene clusters from strains GCUPA1, GCUPA3, and *P. marcusii* CP157, generated using Easyfig. Gray shading indicates nucleotide sequence identity (> 83%). Gene arrows are color-coded according to their biosynthetic pathway features. (**B**) Proposed astaxanthin biosynthetic pathway reconstructed using KEGG Mapper. The pathway is initiated by the isoprenoid precursors isopentenyl pyrophosphate (IPP) and dimethylallyl pyrophosphate (DMAPP) and proceeds through sequential enzymatic conversions: geranylgeranyl pyrophosphate synthase (*crtE*, red), phytoene synthase (*crtB*, orange), phytoene dehydrogenase (*crtI*, yellow), lycopene β-cyclase (*crtY*, green), β-carotene hydroxylase (*crtZ*, blue), and β-carotene ketolase (*crtW*, purple).

AntiSMASH analysis revealed distinct secondary metabolite biosynthetic potential in *P. marcusii* GCUPA1 and GCUPA3, with nine and seven BGCs identified, respectively (Table 4). The GCUPA1 chromosome harbored six BGCs with diverse metabolic capabilities: beta-lactone, the hybrid ectoine/NI-siderophore cluster, homoserine lactone (HSL), the redox cofactor, type 3 polyketide synthase (T3PKS), and terpene biosynthesis. Two additional BGCs were located on the plasmids: a hybrid T1PKS/NRPS/HSL cluster on plasmid pGCUPA1_4 (74.4 kb), and an independent HSL cluster on plasmid pGCUPA1_6. In contrast, strain GCUPA3 contained six chromosomal BGCs representing similar functional categories but lacked the plasmid-encoded T1PKS/NRPS hybrid cluster, consistent with its reduced plasmid content. The terpene BGC present in both strains was identified as a complete AST synthetic cluster that spans 23.6 kb and contains all the essential carotenoid biosynthesis genes (*crtWZYIBE* and *crtX*) (Fig. 9). KEGG pathway analysis confirmed seven enzymatic steps required for AST biosynthesis from the isoprenoid precursors: GGPP synthase (*crtE*), phytoene synthase (*crtB*), phytoene desaturase (*crtI*), lycopene β-cyclase (*crtY*), β-carotene hydroxylase (*crtZ*), β-carotene ketolase (*crtW*), and carotenoid 3,4-desaturase (*crtX*) (Table S4). This gene cluster exhibited complete nucleotide identity with GCUPA1 and GCUPA3, confirming their conserved AST production ability. However, syntenic comparison with the reference strain CP157 revealed different gene arrangements, suggesting independent horizontal gene transfer.

The hybrid ectoine/NI-siderophore cluster demonstrated >95% sequence similarity with homologous clusters from strains CP157, 228, and Arc7-R13, indicating functional conservation within the genus. This cluster encodes enzymes for compatible solute production (ectoine) and iron acquisition (siderophore), both of which are critical for adaptation to environmental stress. The plasmid-borne hybrid cluster in GCUPA1, comprising T1PKS (six modules), NRPS (four modules), and HSL components, exhibited 82% similarity to the cluster in strain CP157. This hybrid functional cluster suggests the potential for complex polyketide-non-ribosomal peptide production, thereby expanding the metabolic diversity of GCUPA1. The presence of three HSL clusters in GCUPA1, as compared to GCUPA3, indicated a robust quorum-sensing capacity in the former representative strain. The expanded BGC content of GCUPA1, particularly in the plasmid-encoded T1PKS/NRPS hybrid cluster, suggests greater biosynthetic versatility that may affect pigment yield and bioactivity profiles.

## Discussion

This initial characterization of AST production in *P. marcusii* strains isolated from freshwater environments has significantly expanded our understanding of bacterial carotenoid biosynthesis beyond the predominant marine and soil habitats [22, 23, 53]. Spectroscopic and chromatographic analyses of the isolated pigment-producing strains GCUPA1 and GCUPA3 from the Geumgang River confirmed AST as the primary carotenoid and established these freshwater isolates as potential alternatives to the existing bacterial production systems. Both strains demonstrated optimal growth at 25°C, which was consistent with the cultivation conditions reported for other carotenoid-producing *P. carotinifaciens* and *P. haeundaensis* [14, 53]. However, the distinct responses of the studied strains to carbon supplementation revealed significant metabolic heterogeneity within this species. GCUPA1 exhibited growth with most of the simple sugars tested, although the growth was reduced compared to the control. In contrast, GCUPA3 exhibited notable growth with only three of the nine carbon sources examined. Notably, both strains demonstrated considerable growth enhancement when supplemented with starch, despite testing negative for carbohydrate fermentation in the API 20E assays. This apparent discrepancy between the two assay systems can be attributed to differences; the API 20E system specifically detects anaerobic fermentative acid production, whereas the carbon source utilization experiments measured growth under aerobic culture conditions. As obligate aerobic Alphaproteobacteria, *Paracoccus* species metabolize carbon sources through oxidative pathways rather than fermentation [54]. In addition, the growth enhancement observed with starch supplementation may suggest the presence of extracellular hydrolytic enzymes that are not detected by biochemical tests, which may involve starch-degrading amylases that function independently of the classical fermentation pathways [55]. Several *Paracoccus* species have been used as probiotics in various fermentation processes, including nitric oxide reduction [56], isopropanol degradation [57], and whey fermentation [58]. The absence of hemolytic activity in strains GCUPA1 and GCUPA3 contrasted markedly with that in *Paracoccus* sp. CP157 harbors genetic determinants like α-hemolysin and adenylate-cyclase toxin [59]. Cytotoxicity assays revealed minimal effects on human HT-1080 cells, with viability maintained above 83%, even at a bacterial concentration of 10 CFU/ml. Both strains exhibited selective antibacterial activity against the marine pathogen *V. alginolyticus*, consistent with the protective effects of *P. marcusii* ND07 against *V. vulnificus* infection in whiteleg shrimp [6], suggesting its potential application in aquaculture feed supplementation. These properties render both strains promising candidates for biosafety applications.

Optimization of the pigment extraction identified acetone as the most effective solvent for carotenoid recovery, yielding 64.1 ± 2.8 μg/g a dry cell weight of GCUPA1 and 45.2 ± 20.1 μg/g GCUPA3. Although these yields represent approximately <1% of the dry biomass, which is substantially lower than the 4% reported for *H. pluvialis* [60], direct comparison of the two strains requires consideration of the utilized bacterial production system. Complete acetone extraction resulted in colorless cell pellets, whereas DMSO-treated cells retained visible pigmentation, indicating incomplete extraction. Notably, *P. marcusii* strains achieved maximal pigment accumulation over 2 or 3 days of cultivation based on our analysis (Fig. S3), which is consistent with the rapid carotenoid production reported for other *P. carotinifaciens* (2-3days) [61] and *P. haeundaensis* (48-72 h) [53]. This rapid production cycle represents a significant advantage compared to the 30-day cultivation cycle required for *H. pluvialis* [62, 63], which may indicate lower yields per cell due to increased production cycles and simplified processing systems. UV-visible spectroscopy revealed an absorption maximum at 465 nm for both strains with a hypsochromic shift in the 470–480 nm range, which is characteristic of purified AST [64]. This spectral absorption feature suggests the presence of AST derivatives in the crude extracts, which is consistent with previously reported carotenoid-modified enzymes, including zeaxanthin glucosyltransferase [6]. HPLC analysis indicated that AST was the predominant carotenoid, with retention times of 16.54 min for GCUPA1 and 16.57 min for GCUPA3, corresponding closely to the authentic free AST standard (16.41 min). The minor deviation in the RT likely reflects matrix interference from the co-extracted compounds rather than structural modifications of AST, as no additional peaks were observed at 480 nm. It should be noted that *Paracoccus* species have been previously reported to produce predominantly free (non-esterified) astaxanthin [61], in contrast to microalgae such as *H. pluvialis*, which primarily accumulate esterified forms [62]. To further evaluate the industrial viability of these strains, we estimated the space-time yield (productivity) compared to conventional microalgal systems. Based on our cultivation data, *P. marcusii* GCUPA1 achieved an AST productivity of approximately 32 μg/l/day under non-optimized conditions, whereas GCUPA3 yielded approximately 22.6 μg/l/day. Although these values are lower than the reported productivity of optimized *H. pluvialis* systems (0.5–2.0 mg/l/day) [62], several factors favor bacterial production systems: i) the 2-day cultivation cycle represents a 15-fold reduction in production time; ii) bacterial systems requires simpler cultivation infrastructure without the need for specialized photobioreactors; iii) downstream processing is facilitated due to the absence of rigid cell walls; and iv) year-round production is feasible without dependence on light availability. With further optimization of culture conditions, carbon sources, and extraction methods, significant improvements in bacterial AST productivity are anticipated.

Genomic analysis revealed genetic content differences between strains, with GCUPA1 harboring 4,046,018 bp and eight plasmids, and GCUPA3 containing 3,446,361 bp and five plasmids. The additional 547 plasmid-encoded genes in GCUPA1 represent functions that are critical for energy metabolism, transport systems, and the stress response, while potentially exhibiting the observed phenotypic variations in growth characteristics and carotenoid yields in the strains. This genomic plasticity has been documented in *P. marcusii* strain CP157, which contains 22 extrachromosomal elements that enhance rapid environmental adaptation [59, 66]. Functional annotation revealed that amino acid and carbohydrate metabolism genes constitute the predominant categories in both genomes, although a substantial proportion of the coding sequences remain functionally uncharacterized, which is a limitation of the proteomic and genomic studies on the genus *Paracoccus* [67]. Nevertheless, the abundance of genes encoding diverse catabolic pathways, including complete carbon degradation systems, indicates the metabolic versatility demonstrated in the phenotypic analyses of both strains. Resistance to piperacillin/tazobactam, which was observed in both strains, appeared to be intrinsic rather than acquired, as no corresponding resistance genes were detected in the CARD analysis. Similar intrinsic β-lactam resistance has been reported in other non-pathogenic *Paracoccus* species and is considered a genus-level characteristic rather than an indicator of acquired resistance potential [68, 69].

Virulence factor analysis identified only housekeeping functions, with flagellar biosynthesis components (*flhA*, *flmH*) and a type VI secretion element (*clpV*) that are widely distributed in environmental bacteria and lack the adhesins, invasins, and toxin systems that are characteristic of pathogens [70, 71]. AntiSMASH analysis predicted T3PKs and beta-lactone clusters for both strains, which are biosynthetic systems known to generate structurally diverse antimicrobials in other bacterial taxa [59]. T3PKS products demonstrate broad-spectrum antibacterial activity via the disruption of fatty acid biosynthesis [72, 73], whereas β-lactones function as serine hydrolase inhibitors with documented anti-virulence properties against gram-negative pathogens [74]. Additionally, the identification of bacteriocins and hybrid PKS/NRPS clusters revealed architectural features that are commonly associated with narrow-spectrum antimicrobials in *Paracoccus* species [59]. The potential quorum-sensing machinery, with GCUPA1 harboring three homoserine lactone synthase clusters as compared to the two observed in GCUPA3, suggests the strain-specific regulation of secondary metabolism through cell density-dependent signals [75, 76]. Beyond secondary metabolite production, competitive exclusion mechanisms, including nutrient competition and niche occupation, may also contribute to the observed anti-*Vibrio* activity [77-79]. Accordingly, further biochemical characterization of culture supernatants and purified metabolites will be necessary to definitively identify the active antimicrobial compounds.

Genome-based phylogeny showed GCUPA1 and GCUPA3 within a well-supported monophyletic clade of *P. marcusii* strains, with OrthoANI values >97%, confirming species designation. The identification of 3,061 core genes representing 78% of the total gene content indicates substantial genomic conservation within the species, whereas strain-specific variation in genes suggests environmental adaptation [40]. The absence of strain-specific gene clusters in GCUPA3, in contrast with the 18 unique clusters in GCUPA1, may reflect different determinants, such as horizontal gene transfer, which has been observed previously in these genomes [67]. The AST biosynthetic gene cluster, which encodes all seven essential enzymes from isoprenoid precursors to AST, indicates complete nucleotide identity between strains GCUPA1 and GCUPA3. This syntenic organization is maintained despite the distinct plasmid profiles and metabolic characteristics, as indicated by KEGG pathway analysis, which suggested a conserved enzyme function for *Paracoccus* [15]. Notably, the astaxanthin BGC (terpene BGC harboring *crtWZYIBE* and *crtX* genes) in strains GCUPA1 and GCUPA3 was localized on the chromosome, not on plasmids. Chromosomal encoding of the astaxanthin biosynthetic pathway confers greater genetic stability compared to plasmid-borne gene clusters, as chromosomal genes are less prone to segregational loss during in the absence of selective pressure [80]. The conserved chromosomal localization and complete nucleotide identity (100%) of the astaxanthin BGC between two strains, combined with the distinct gene arrangements compared to the reference strain CP157 [59]. The presence of additional BGCs, including the plasmid-borne T1PKS/NRPS hybrid cluster unique to GCUPA1, suggests broader secondary metabolite production capabilities.

The presence of additional BGCs, including the plasmid-borne T1PKS/NRPS hybrid cluster unique to GCUPA1, suggests broader secondary metabolite production capabilities that may contribute to the overall cellular metabolism and pigment yields. Similar PKS and NRPS clusters have been identified as the most abundant BGCs in *Paracoccus* species, with products ranging from siderophores to novel antimicrobial compounds [81]. The *pqqABCDE* operon identified in both strains represents a unique feature rarely reported in AST-producing bacteria [82] and may contribute to enhanced oxidative metabolism and stress tolerance during carotenoid production.

The observed genetic and phenotypic antibacterial effects indicate that the studied strains are promising agents and microbial sources of colorants. Genomic analyses provided unprecedented insights into the biosynthetic capabilities and safety profiles of both strains.

## Conclusion

The present study established *P. marcusii* strains GCUPA1 and GCUPA3 as promising candidates for industrial AST production. The two strains demonstrate efficient pigment biosynthesis with yields of 64.1 μg/g and 45.2 μg/g dry cell weight, respectively, and the rapid cultivation period of only two days represents a significant advancement over the 30-day cycle required for *H. pluvialis*; however, the increased production cycles and simplified extraction processes may potentially result in lower volumetric yields. Complete genomic characterization confirmed the presence of intact carotenoid BGCs encoding all the essential enzymes (*crtWZYIBE*) required for AST synthesis, and safety assessments revealed no clinically relevant antibiotic resistance genes or virulence factors. The observed resistance to piperacillin/tazobactam appears to be intrinsic to the genus rather than acquired. Moreover, both strains exhibited selective antibacterial activity against *V. alginolyticus* at 10^7^ CFU/ml under the tested conditions, and cytotoxicity evaluation using the WST-8 assay demonstrated minimal effects on human HT-1080 cells at concentrations up to 10^7^ CFU/ml, supporting their potential applicability as natural antimicrobial agents for aquaculture. The results of this study are expected to provide a foundation for the establishment of *P. marcusii* as a sustainable candidate for the production of natural colorants, highlighting the biosafety and broad biotechnological potential of the studied strains.

## Supplemental Materials

Supplementary data for this paper are available on-line only at http://jmb.or.kr.



## Figures and Tables

**Table 1:** Antimicrobial susceptibility profiles of *P. marcusii* GCUPA1 and GCUPA3 determined by the minimum inhibitory concentration (MIC) test.

Class	Antibiotics	Concentration (μg/ml)		MIC (μg/ml)	
		From	To	GCUPA1	GCUPA3
Aminoglycoside	Amikacin^a^	0.015	256	1.5	0.75
	Gentamicin^a^	0.06	1024	0.5	0.19
	Tobramycin^a^	0.064	1024	0.5	0.19
Cephalosporin	Ceftazidime^b^	0.016	256	1.5	2
Carbapenem	Imipenem^b^	0.002	32	0.125	0.094
	Meropenem^b^	0.002	32	0.016	0.023
Penicillin/beta-lactamase inhibitor	Piperacillin/tazobactam^b^	0.016/4	256/4	256/4	256/4
Fluoroquinolone	Ciprofloxacin^b^	0.002	32	0.125	0.016

MIC values were obtained using gradient diffusion strips on Mueller-Hinton agar following 48 h incubation at 25°C. Susceptibility was based on clinical breakpoints for *Pseudomonas* spp. and *Acinetobacter* spp. or non-species-related pharmacokinetic/pharmacodynamic (PK-PD) breakpoints from the European Committee on Antimicrobial Susceptibility Testing (EUCAST) guidelines. The category of antibiotic susceptibility is indicated as follows: Gray, resistant; white, susceptible. The results of MIC evaluations are shown in number.

^a^*Pseudomonas* and *Acinetobacter* spp. breakpoints

^b^PK-PD (Pharmacokinetics−Pharmacodynamics; Non-species related) breakpoints

**Table 2:** Antibacterial activity of *P. marcusii* GCUPA1 and GCUPA3 against eight reference bacterial strains.

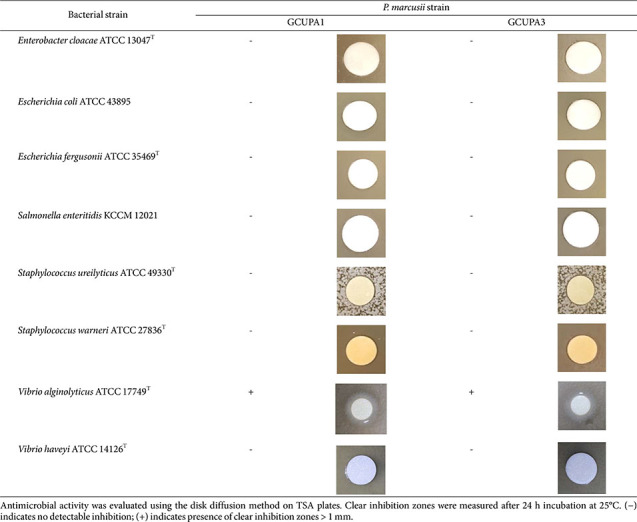

**Table 3:** Comparative analysis of carotenoid extraction efficiency for *P. marcusii* GCUPA1 and GCUPA3 using different organic solvents.

Strain	Solvent	TCC (μg/g)
GCUPA1	Acetone	64.1 ± 2.9
	Methanol	26.7 ± 1.7
	DMSO	33.5 ± 0.9
GCUPA3	Acetone	45.2 ± 20.1
	Methanol	19.5 ± 5.7
	DMSO	22.6 ± 2.2

Total carotenoid content (TCC) was determined spectrophotometrically at 480 nm using solvent-specific absorption coefficients (${\text{A}}_{\text{1cm}}^{\text{1\%}}$), with 2100 for acetone and methanol, 2220 for DMSO. Values represent mean TCC (μg/g dry cell weight) ± standard deviation from three independent extractions.

**Table 4:** Secondary metabolite biosynthesis gene clusters (BGCs) identified in *P. marcusii* GCUPA1 and GCUPA3 genomes using antibiotics and secondary metabolite analysis using antiSMASH.

Strain	Location	Position (bp)		Type^a^	Biosynthesis gene cluster	Substance	Similarity^b^ (%)
		From	To				
GCUPA1	Chromosome	436,039	461,979	Betalactone	Corynecin III/corynecin I/corynecin II	Corynecin	13
		1,112,027	1,149,285	Hybrid (ectoine,NI-siderophore)	Ectoine	Ectonine	100
		2,043,529	2,064,119	Hserlactone	N.D.	N.D.	
		2,652,653	2,674,828	Redox-cofactor	N.D.	N.D.	
		2,997,797	3,038,855	T3PKS	N.D.	N.D.	
		3,050,285	3,073,858	Terpene	Terpene	Astaxanthin	100
	Plasmid 3	1	67,314	Hybrid (T1PKS, NRPS-like, NRPS, hserlactone)	N.D.	N.D.	
		114,620	135,861	RRE-containing	N.D.	N.D.	
		206,518	217,363	RiPP-like	N.D.	N.D.	
	Plasmid 5	977	21,606	Hserlactone	N.D.	N.D.	
GCUPA3	Chromosome	186,462	227,520	T3PKS	N.D.	N.D.	
		238,950	262,523	Terpene	Carotenoid	Astaxanthin	100
		698,048	723,988	Betalactone	Corynecin III/corynecin I/corynecin II	Corynecin	13
		1,374,036	1,411,294	Hybrid (ectoine, NI-siderophore)	Ectoine	Ectoine	100
		2,305,543	2,326,133	Hserlactone	N.D.	N.D.	
	Plasmid 2	35,515	48,823	Hserlactone	N.D.	N.D.	

T1PKS refers to type 1 polyketide synthase; T3PKS, type 3 polyketide synthase; NRPS, non-ribosomal peptide synthetase; RRE, RiPP recognition element; RiPP, ribosomally synthesized and post-translationally modified peptides; ND, not determined.

^a^Based on the MIBiG database

^b^Similarity to the closest BGC in the antiSMASH/MIBiG database
