# Supplementary material for: Biological and Genomic Characterization of Two Astaxanthin-Producing Paracoccus marcusii Isolates as a Potential Source for Food Additives
Source: J Microbiol Biotechnol. 2026 Mar 26;36:e2512023. doi: 10.4014/jmb.2512.12023 (PMC13036509; doi:10.4014/jmb.2512.12023)
Supplement: Supplementary file 1 [file jmb-36-e2512023-supple.pdf]

J. Microbiol. Biotechnol. 2026. 36: e2512023

https://doi.org/10.4014/jmb.2512.12023

Supplementary Tables and Figures

Table S1. Biochemical characteristics of *P. marcusii* GCUPA1 and GCUPA3 determined using the API 20E identification system.

Strain	ONPG	ADH	LDC	ODC	CIT	H2S	URE	TDA	IND	VP	GEL	GLU	MAN	INO	SOR	RHA	SAC	MEL	AMY	ARA
GCUPA1	+	-	-	-	-	-	-	-	+	-	-	-	-	-	-	-	-	-	-	-
GCUPA3	+	-	-	-	-	-	-	-	+	-	-	-	-	-	-	-	-	-	-	-

ONPG, β-galactosidase; ADH, arginine dihydrolase; LDC, lysine decarboxylase; ODC, ornithine decarboxylase; CIT, citrate utilization; H2S, H_2S production; URE, urease; TDA, tryptophane deaminase; IND, indole production; VP, acetoin production; GEL, gelatinase; GLU, glucose; MAN, mannitol; INO, inositol; SOR, sorbitol; RHA, rhamnose; SAC, saccharose; MEL, melibiose; AMY, amygdalin; ARA, arabinose.

Table S2. **Genomic features of *P. marcusii* strain GCUPA1 and GCUPA3**.

Features	GCUPA1	GCUPA3
Length (bp)	4,046,018	3,446,361
G + C contents (%)	66.6	67.3
No. of Plasmid	8	5
CDS	3,922	3,335
Pseudogene	49	16
tRNA	52	50
rRNA	9	9
ncRNA	3	3

Table S3. Virulence factor analysis of *P. marcusii* GCUPA1 and GCUPA3 genomes using the Virulence Factor Database (VFDB).

Strain	Location	VFclass[a]	Virulence Factor	Gene	Position(bp)	
					From	To
GCUPA1	Chromosome	Adherence	Polar flagella	*flhA*	2,525,321	2,527,420
				flmH	2,167,903	2,168,640
		Secretion system	T6SS	*clpV1*	1,390,128	1,392,452
GCUPA3	Chromosome	Adherence	Polar flagella	*flhA*	2,787,335	2,789,434
				flmH	2,429,917	2,430,654
		Secretion system	T6SS	*clpV1*	1,652,137	1,654,461

[a] Potential virulence-associated genes were identified through homology searches against the Virulence Factor Database (VFDB) using *Vibrio* spp. as the reference dataset. Only genes with >40% amino acid identity and >60% query coverage were considered significant.

Table S4. Prophage regions detected in *P. marcusii* GCUPA1 and GCUPA3 genomes using the PHAge Search Tool with Enhanced Sequence Translation (PHASTEST).

Strain	Location	Position		Size (kb)	Score[a]	#[b]	Most Common Phage (Accession number; number of proteins)[c]	GC %
		From	To					
GCUPA1	Chromosome	5,047	24,288	19.2	90	21	Salico phage CGphi29 (NC 020844; 4)	65.3
		48,127	67,371	19.2	100	22	Salico phage CGphi29 (NC 020844; 4)	65.3
		695,520	710,654	15.1	110	18	Dinoro phage vB_DshS_R5C (NC 041921; 5)	68
		963,750	978,066	14.3	80	16	Brucel phage BiPBO1 (NC 031264; 2)	67.1
GCUPA3	Chromosome	310,133	329,378	19.2	100	21	Salico phage CGphi29 (NC 020844; 4)	65.3
		957,529	972,663	15.1	110	18	Dinoro phage vB_DshS_R5C (NC 041921; 5)	68
		1,225,759	1,240,075	14.3	80	16	Phage Gifsy 2 (NC 010393; 2)	67.1

[a] Score based on PHASTEST criteria (>90, intact; >70, questionable; >0, incomplete)

[b] Number of proteins in the region

[c] The name and accession number of phage with the highest number of proteins in the region (between parentheses: number of proteins).

Table S5. Astaxanthin biosynthetic pathway genes identified in *P. marcusii* GCUPA1 and GCUPA3 genomes using BlastKOALA against the KEGG database, followed by pathway construction using KEGG Mapper.

Strain	location	Position (bp)		KEGG gene name	Gene annotation[a]
		From	to		
GCUPA1	Chromosome	3,059,075	3,059,803	*crtW; BKT*	beta-carotene/zeaxanthin 4-ketolase
		3,059,800	3,060,288	*crtZ*	beta-carotene 3-hydroxylase
		3,060,285	3,061,445	*lcyB; crtL1; crtY*	lycopene beta-cyclase
		3,061,442	3,062,947	*crtI*	phytoene desaturase
		3,062,944	3,063,858	*crtB*	15-cis-phytoene synthase
		3,063,855	3,064,736	*ispA; crtE*	farnesyl diphosphate synthase
GCUPA3	Chromosome	247,740	248,468	*crtW; BKT*	beta-carotene/zeaxanthin 4-ketolase
		248,465	248,953	*crtZ*	beta-carotene 3-hydroxylase
		248,950	250,110	*lcyB; crtL1; crtY*	lycopene beta-cyclase
		250,107	251,612	*crtI*	phytoene desaturase
		251,609	252,523	*crtB*	15-cis-phytoene synthase
		252,520	253,401	*ispA; crtE*	farnesyl diphosphate synthase

[a] Listed genes encode the complete enzymatic machinery for astaxanthin biosynthesis from isoprenoid precursors.

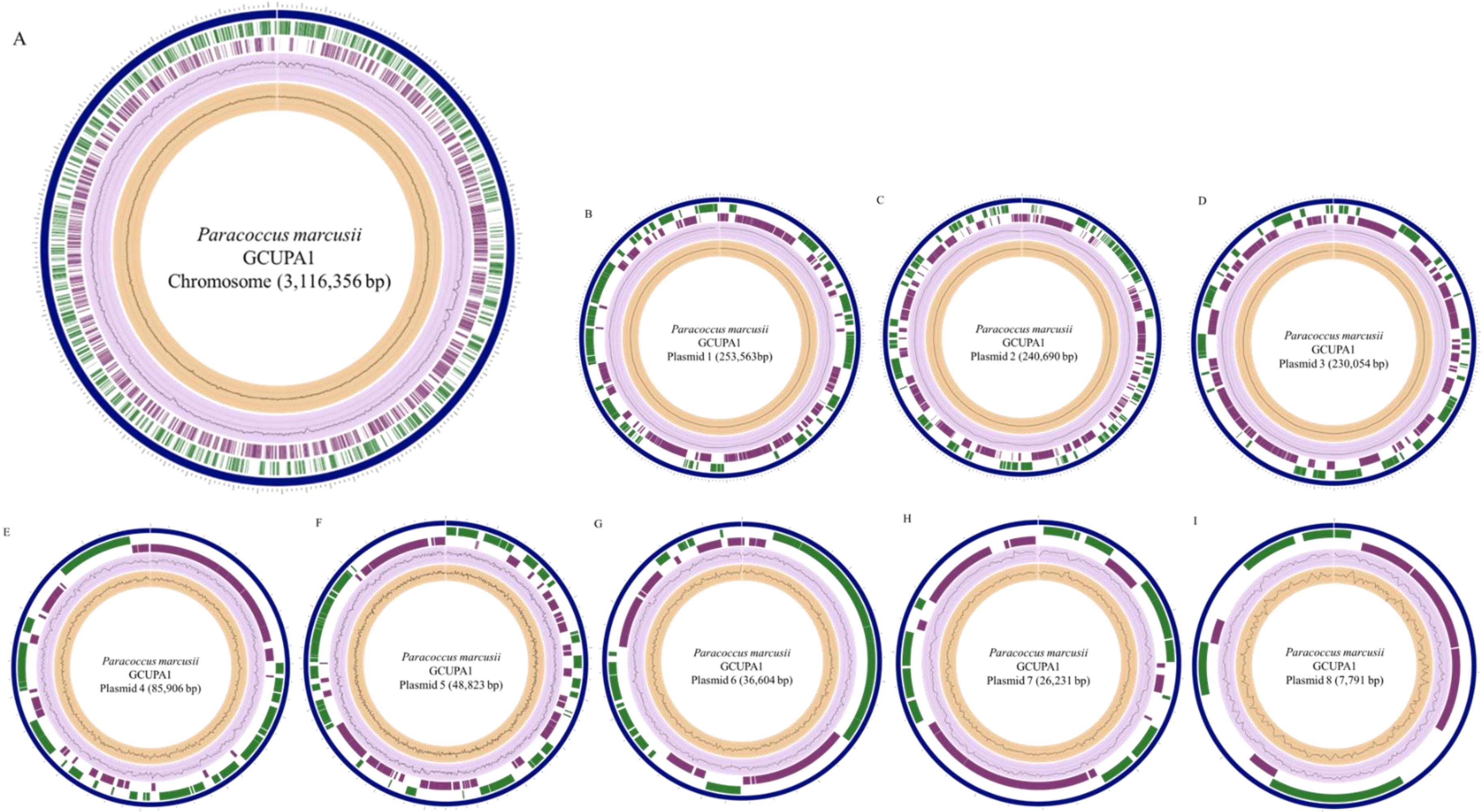
A
Paracoccus marcusii
GCUPA1
Chromosome (3,116,356 bp)
B
Paracoccus marcusii
GCUPA1
Plasmid 1 (253,563bp)
C
Paracoccus marcusii
GCUPA1
Plasmid 2 (240,690 bp)
D
Paracoccus marcusii
GCUPA1
Plasmid 3 (230,054 bp)
E
Paracoccus marcusii
GCUPA1
Plasmid 4 (85,906 bp)
F
Paracoccus marcusii
GCUPA1
Plasmid 5 (48,823 bp)
G
Paracoccus marcusii
GCUPA1
Plasmid 6 (36,604 bp)
H
Paracoccus marcusii
GCUPA1
Plasmid 7 (26,231 bp)
I
Paracoccus marcusii
GCUPA1
Plasmid 8 (7,791 bp)

Fig. S1. **Circular genome maps of *P. marcusii* GCUPA1 visualized using the PATRIC genome annotation pipeline on the BV-BRC platform (A–I).** Complete genome of strain GCUPA1 comprising a chromosome **(A)** and eight plasmids **(B–I).** Circular representations display, from outer to inner rings: (i) forward strand CDS (green), (ii) reverse strand CDS (purple), (iii) GC content (black), and (iv) GC skew (orange).

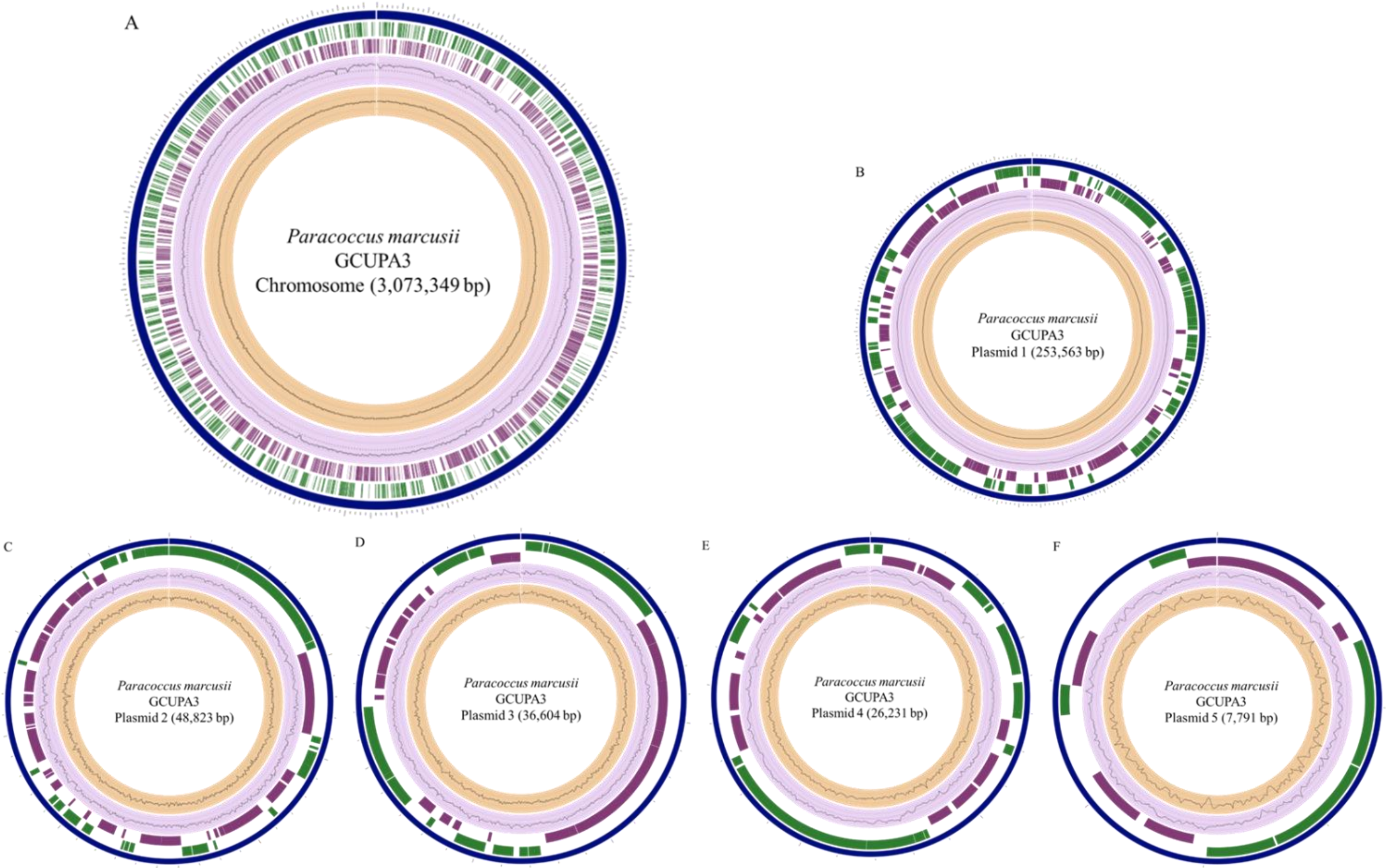
A
Paracoccus marcusii
GCUPA3
Chromosome (3,073,349 bp)
B
Paracoccus marcusii
GCUPA3
Plasmid 1 (253,563 bp)
C
Paracoccus marcusii
GCUPA3
Plasmid 2 (48,823 bp)
D
Paracoccus marcusii
GCUPA3
Plasmid 3 (36,604 bp)
E
Paracoccus marcusii
GCUPA3
Plasmid 4 (26,231 bp)
F
Paracoccus marcusii
GCUPA3
Plasmid 5 (7,791 bp)

Fig. S2. **Circular genome maps of *P. marcusii* GCUPA3 visualized using the PATRIC genome annotation pipeline on the BV-BRC platform (A–F).** Complete genome of strain GCUPA3, consisting of a chromosome **(A)** and five plasmids **(B–F)**. Circular representations display, from outer to inner rings: (i) forward strand CDS (green), (ii) reverse strand CDS (purple), (iii) GC content (black), and (iv) GC skew (orange).

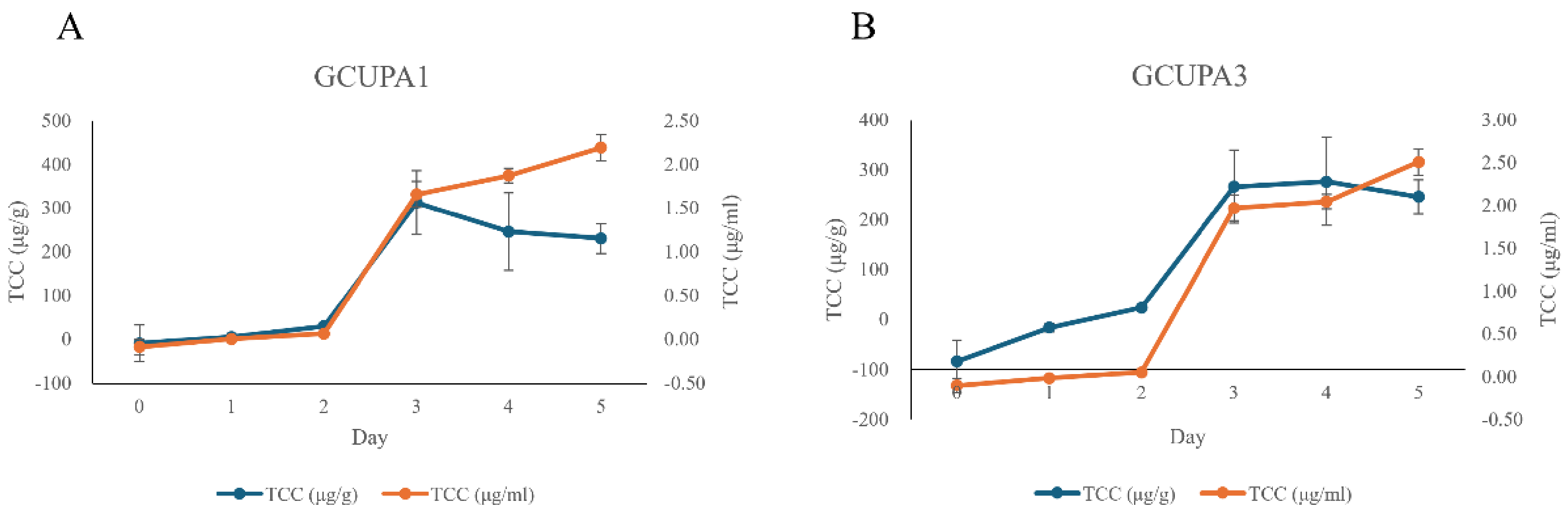

Fig. S3. Changes in TCC content in GCUPA1 (A) and GCUPA3 (B). The blue line indicates pigment content (µg g^{-1} biomass), whereas the orange line indicates pigment content (µg mL^{-1} culture broth).
